# (*S*)-2-Oxotetra­hydro­furan-3-aminium bromide^1^

**DOI:** 10.1107/S1600536812032552

**Published:** 2012-07-25

**Authors:** Jace D. Sandifer, Frank R. Fronczek, Steven F. Watkins

**Affiliations:** a Department of Chemistry, Louisiana State University, Baton Rouge, LA 70803-1804, USA

## Abstract

In the title HBr salt of (*S*)-homoserine lactone, C_4_H_8_NO_2_^+^·Br^−^, the five-membered ring has an envelope conformation, with the –CH_2_– C atom adjacent to the N-substituted C atom at the flap position. The four-atom mean plane (r.m.s. deviation = 0.005 Å) of the envelope forms a dihedral angle of 32.12 (9)° with the three-atom flap plane. The distorted square-pyramidal coordination about the anion involves five surrounding cations, with the square base defined by three N—H⋯Br hydrogen bonds [Br⋯N = 3.3046 (10), 3.3407 (12) and 3.3644 (13) Å] and near-contact with an H atom attached to C [Br⋯C = 3.739 (1) Å]. Another Br⋯C contact of 3.427 (1) Å defines the apex. There is also an N—H⋯O hydrogen bond present linking the cations.

## Related literature

For related crystal structures, see: Bocelli & Grenier-Loustalot (1981 ▶); Papaioannou *et al.* (1990 ▶). For the synthesis of the title compound, see: Armstrong (1948 ▶); Cowell (1996 ▶). For the Cambridge Structural Database, see: Allen (2002 ▶).
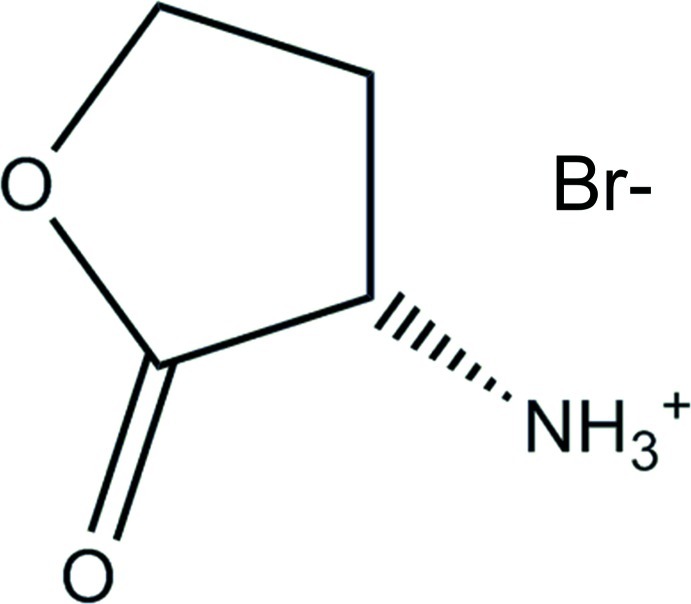


## Experimental

### 

#### Crystal data

C_4_H_8_NO_2_^+^·Br^−^
*M* *_r_* = 182.02
Orthorhombic, 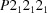

*a* = 6.1425 (1) Å
*b* = 9.4196 (2) Å
*c* = 11.0394 (3) Å
*V* = 638.74 (2) Å^3^
*Z* = 4
Mo *K*α radiation
μ = 6.35 mm^−1^
*T* = 90 K
0.25 × 0.25 × 0.22 mm

#### Data collection

Nonius KappaCCD diffractometer
Absorption correction: multi-scan (*SCALEPACK*; Otwinowski & Minor, 1997 ▶) *T*_min_ = 0.300, *T*_max_ = 0.336
4072 measured reflections
4072 independent reflections
3915 reflections with *I* > 2σ(*I*)
*R*_int_ = 0

#### Refinement

*R*[*F* ^2^ > 2σ(*F* ^2^)] = 0.022
*wR*(*F* ^2^) = 0.049
*S* = 1.07
4072 reflections
76 parameters
H-atom parameters constrained
Δρ_max_ = 0.68 e Å^−3^
Δρ_min_ = −0.74 e Å^−3^
Absolute structure: Flack (1983 ▶) and Hooft *et al.* (2008 ▶), with 1724 Friedel pairs
Flack parameter: 0.030 (7)

### 

Data collection: *COLLECT* (Nonius, 2000 ▶); cell refinement: *SCALEPACK* (Otwinowski & Minor, 1997 ▶); data reduction: *DENZO* (Otwinowski & Minor, 1997 ▶) and *SCALEPACK*; program(s) used to solve structure: *SIR2002* (Burla *et al.*, 2003 ▶); program(s) used to refine structure: *SHELXL97* (Sheldrick, 2008 ▶); molecular graphics: *ORTEP-3 for Windows* (Farrugia, 1997 ▶); software used to prepare material for publication: *IDEAL* (Gould *et al.*, 1988 ▶) and *WinGX* (Farrugia, 1999 ▶).

## Supplementary Material

Crystal structure: contains datablock(s) global, I. DOI: 10.1107/S1600536812032552/zq2176sup1.cif

Structure factors: contains datablock(s) I. DOI: 10.1107/S1600536812032552/zq2176Isup2.hkl

Supplementary material file. DOI: 10.1107/S1600536812032552/zq2176Isup3.cml

Additional supplementary materials:  crystallographic information; 3D view; checkCIF report

## Figures and Tables

**Table 1:** Hydrogen-bond geometry (Å, °)

*D*—H⋯*A*	*D*—H	H⋯*A*	*D*⋯*A*	*D*—H⋯*A*
N1—H1*A*⋯Br1^i^	0.91	2.47	3.3407 (12)	161
N1—H1*B*⋯Br1^ii^	0.91	2.41	3.3046 (10)	168
N1—H1*C*⋯Br1^iii^	0.91	2.51	3.3644 (13)	157
N1—H1*C*⋯O1^iii^	0.91	2.5	3.0050 (14)	115

Symmetry codes: (i) 
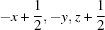
; (ii) 
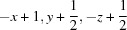
; (iii) 
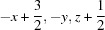
.

**Fig. 1.**
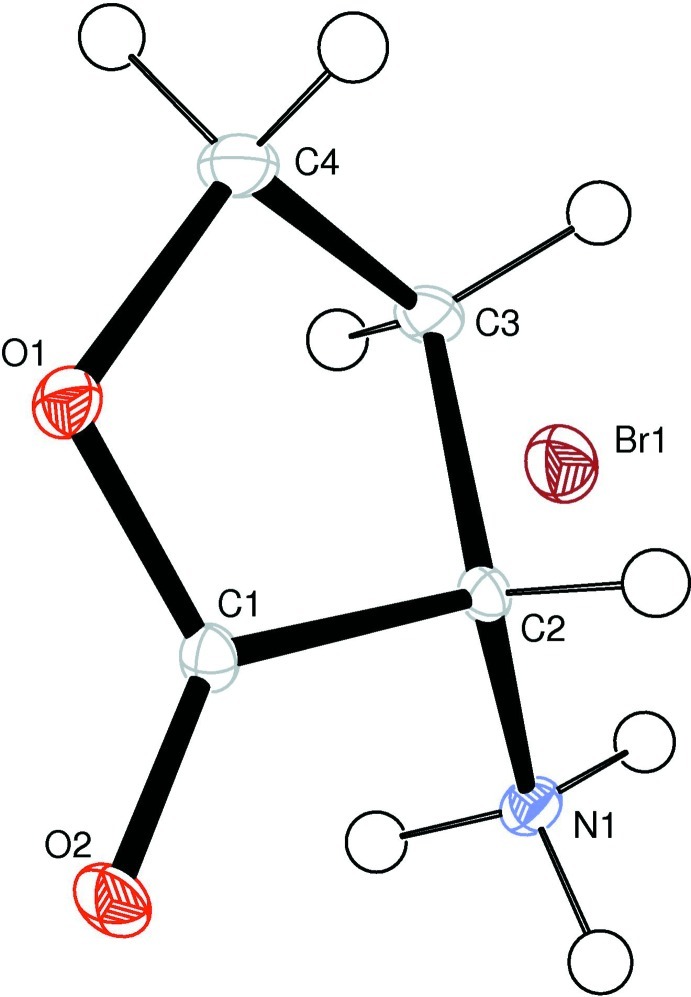
View of the title compound (50% probability displacement ellipsoids)

## supplementary crystallographic information

### Comment

The structure of the racemate of title compound I was determined at 295 K by Bocelli & Grenier-Loustalot (1981; BAKHAW, Allen, 2002); the geometries of the cation in BAKHAW and I are similar, as characterized by least-squares fit of all non-hydrogen atoms in the cation (δ_r.m.s._ = 0.025; Gould *et al.*, 1988). In addition, the envelope flap angles are similar (31.0° *versus* 32.6°). The racemate at 295 K has a volume per formula unit of 162.3 Å^3^, whereas pure enantiomer I at 90 K has a volume per formula unit of 159.7 Å^3^, a decrease of about 1.6%.

The chloride analog of I (TADTAT; Papaioannou *et al.*, 1990) crystallizes in the same space group as I and with similar lattice constants, but the geometries of the cation differ slightly (δ_r.m.s._ = 0.094), and the envelope flap angles also differ: 17.58° *versus* 32.6°.

There are no intramolecular H-bonds in I. However, all three H-atoms of the ammonium group participate in intermolecular H-bonding to form a three-dimensional network. These H atoms bond to three different anions [N···Br = 3.3046 (10), 3.3407 (12) and 3.3644 (13) Å] and to an ether oxygen: N···O1 = 3.0050 (14) Å.

### Experimental

The earliest preparation of racemic and enantiomerically pure homoserine lactones was reported by Armstrong (1948). Cowell recrystallized I from methanol and provided the crystal used for data collection (Cowell, 1996).

### Refinement

Absolute configuration was determined by analysis of 1724 Bijvoet pairs: Flack (Flack, 1983) parameter = 0.030 (7), Hooft (Hooft *et al.*, 2008) parameter = 0.035 (5) and P2(true) = 1.00. All H atoms were placed in calculated positions, guided by difference maps, with C—H bond distances 1.00 (C2) and 0.99 (C3, C4), and N—H distances 0.91 Å, with *U*_iso_ = 1.2*U*_eq_ for each C—H and *U*_iso_ = 1.5*U*_eq_ for each N—H, thereafter refined as riding. A torsional parameter for the ammonium group was also refined.

### Crystal data

**Table:**

C_4_H_8_NO_2_^+^·Br^−^	*D*_x_ = 1.893 Mg m^−^^3^
*M**_r_* = 182.02	Melting point: 514 K
Orthorhombic, *P*2_1_2_1_2_1_	Mo *K*α radiation, λ = 0.71073 Å
Hall symbol: P 2ac 2ab	Cell parameters from 2320 reflections
*a* = 6.1425 (1) Å	θ = 2.6–41.2°
*b* = 9.4196 (2) Å	µ = 6.35 mm^−^^1^
*c* = 11.0394 (3) Å	*T* = 90 K
*V* = 638.74 (2) Å^3^	Prism, colourless
*Z* = 4	0.25 × 0.25 × 0.22 mm
*F*(000) = 360	

### Data collection

**Table:**

Nonius KappaCCD diffractometer	4072 independent reflections
Radiation source: sealed tube	3915 reflections with *I* > 2σ(*I*)
Horizonally mounted graphite crystal monochromator	*R*_int_ = 0
Detector resolution: 9 pixels mm^-1^	θ_max_ = 40.8°, θ_min_ = 2.8°
CCD rotation images, thick slices scans	*h* = −11→11
Absorption correction: multi-scan (*SCALEPACK*; Otwinowski & Minor, 1997)	*k* = −16→17
*T*_min_ = 0.300, *T*_max_ = 0.336	*l* = −20→20
4072 measured reflections	

### Refinement

**Table:**

Refinement on *F*^2^	Hydrogen site location: inferred from neighbouring sites
Least-squares matrix: full	H-atom parameters constrained
*R*[*F*^2^ > 2σ(*F*^2^)] = 0.022	*w* = 1/[σ^2^(*F*_o_^2^) + (0.0124*P*)^2^ + 0.6239*P*] where *P* = (*F*_o_^2^ + 2*F*_c_^2^)/3
*wR*(*F*^2^) = 0.049	(Δ/σ)_max_ = 0.001
*S* = 1.07	Δρ_max_ = 0.68 e Å^−^^3^
4072 reflections	Δρ_min_ = −0.74 e Å^−^^3^
76 parameters	Extinction correction: *SHELXL97* (Sheldrick, 2008), Fc^*^=kFc[1+0.001xFc^2^λ^3^/sin(2θ)]^-1/4^
0 restraints	Extinction coefficient: 0.0198 (9)
0 constraints	Absolute structure: Flack (1983) and Hooft *et al.* (2008), with 1724 Friedel pairs
Primary atom site location: structure-invariant direct methods	Flack parameter: 0.030 (7)
Secondary atom site location: difference Fourier map	

### Special details

**Table:**

Geometry. All e.s.d.'s (except the e.s.d. in the dihedral angle between two l.s. planes) are estimated using the full covariance matrix. The cell e.s.d.'s are taken into account individually in the estimation of e.s.d.'s in distances, angles and torsion angles; correlations between e.s.d.'s in cell parameters are only used when they are defined by crystal symmetry. An approximate (isotropic) treatment of cell e.s.d.'s is used for estimating e.s.d.'s involving l.s. planes.

### Fractional atomic coordinates and isotropic or equivalent isotropic displacement parameters (Å^2^)

**Table:**

	*x*	*y*	*z*	*U*_iso_*/*U*_eq_	
Br1	0.398524 (19)	−0.035758 (13)	−0.012904 (11)	0.00918 (3)	
C1	0.73144 (19)	−0.01395 (12)	0.23546 (12)	0.00862 (18)	
C2	0.64286 (18)	0.13091 (12)	0.27008 (11)	0.00776 (17)	
H2	0.505	0.1494	0.2246	0.009*	
C3	0.8183 (2)	0.23236 (13)	0.22670 (12)	0.01110 (19)	
H3A	0.9319	0.2468	0.2891	0.013*	
H3B	0.7564	0.3254	0.2032	0.013*	
C4	0.9071 (3)	0.15234 (14)	0.11731 (11)	0.01289 (19)	
H4A	0.8245	0.1772	0.0433	0.015*	
H4B	1.0626	0.1752	0.1042	0.015*	
N1	0.5994 (2)	0.13823 (11)	0.40235 (9)	0.00966 (14)	
H1A	0.4755	0.0892	0.4197	0.014*	
H1B	0.5828	0.2305	0.4249	0.014*	
H1C	0.7131	0.0994	0.4435	0.014*	
O1	0.87958 (17)	0.00107 (10)	0.14696 (9)	0.01107 (15)	
O2	0.68198 (18)	−0.12734 (10)	0.27816 (10)	0.01255 (16)	

### Atomic displacement parameters (Å^2^)

**Table:**

	*U*^11^	*U*^22^	*U*^33^	*U*^12^	*U*^13^	*U*^23^
Br1	0.00741 (4)	0.00830 (4)	0.01182 (4)	−0.00006 (4)	−0.00068 (3)	0.00165 (3)
C1	0.0080 (4)	0.0070 (5)	0.0109 (4)	−0.0001 (3)	−0.0007 (3)	−0.0011 (3)
C2	0.0082 (4)	0.0052 (4)	0.0099 (4)	0.0006 (3)	0.0002 (3)	−0.0001 (3)
C3	0.0133 (5)	0.0066 (4)	0.0134 (5)	−0.0012 (4)	0.0025 (4)	0.0011 (3)
C4	0.0159 (5)	0.0101 (4)	0.0126 (4)	−0.0012 (5)	0.0040 (5)	0.0010 (3)
N1	0.0093 (3)	0.0084 (3)	0.0112 (4)	−0.0007 (4)	0.0026 (4)	−0.0013 (3)
O1	0.0120 (4)	0.0086 (3)	0.0126 (3)	0.0003 (3)	0.0033 (3)	−0.0017 (3)
O2	0.0132 (4)	0.0063 (3)	0.0181 (4)	−0.0017 (3)	−0.0006 (3)	0.0009 (3)

### Geometric parameters (Å, º)

**Table:**

C1—O2	1.2063 (15)	C3—H3B	0.99
C1—O1	1.3427 (16)	C4—O1	1.4717 (16)
C1—C2	1.5179 (17)	C4—H4A	0.99
C2—N1	1.4861 (16)	C4—H4B	0.99
C2—C3	1.5178 (17)	N1—H1A	0.91
C2—H2	1	N1—H1B	0.91
C3—C4	1.5244 (18)	N1—H1C	0.91
C3—H3A	0.99		
			
O2—C1—O1	123.25 (12)	C4—C3—H3B	111.6
O2—C1—C2	127.40 (12)	H3A—C3—H3B	109.4
O1—C1—C2	109.35 (10)	O1—C4—C3	105.16 (10)
O2—C1—Br1	96.27 (8)	O1—C4—H4A	110.7
O1—C1—Br1	80.12 (7)	C3—C4—H4A	110.7
C2—C1—Br1	92.37 (7)	O1—C4—H4B	110.7
N1—C2—C1	110.70 (10)	C3—C4—H4B	110.7
N1—C2—C3	114.11 (10)	H4A—C4—H4B	108.8
C1—C2—C3	103.42 (10)	C2—N1—H1A	109.5
N1—C2—H2	109.5	C2—N1—H1B	109.5
C1—C2—H2	109.5	H1A—N1—H1B	109.5
C3—C2—H2	109.5	C2—N1—H1C	109.5
C2—C3—C4	101.11 (10)	H1A—N1—H1C	109.5
C2—C3—H3A	111.6	H1B—N1—H1C	109.5
C4—C3—H3A	111.6	C1—O1—C4	109.98 (10)
C2—C3—H3B	111.6		
			
O2—C1—C2—N1	35.80 (17)	C1—C2—C3—C4	31.16 (13)
O1—C1—C2—N1	−144.09 (10)	C2—C3—C4—O1	−31.04 (14)
Br1—C1—C2—N1	135.54 (8)	O2—C1—O1—C4	−178.50 (13)
O2—C1—C2—C3	158.41 (13)	C2—C1—O1—C4	1.40 (14)
O1—C1—C2—C3	−21.48 (13)	Br1—C1—O1—C4	90.45 (9)
Br1—C1—C2—C3	−101.84 (8)	C3—C4—O1—C1	19.30 (15)
N1—C2—C3—C4	151.48 (11)		

### Hydrogen-bond geometry (Å, º)

**Table:**

*D*—H···*A*	*D*—H	H···*A*	*D*···*A*	*D*—H···*A*
N1—H1*A*···Br1^i^	0.91	2.47	3.3407 (12)	161
N1—H1*B*···Br1^ii^	0.91	2.41	3.3046 (10)	168
N1—H1*C*···Br1^iii^	0.91	2.51	3.3644 (13)	157
N1—H1*C*···O1^iii^	0.91	2.5	3.0050 (14)	115

Symmetry codes: (i) −*x*+1/2, −*y*, *z*+1/2; (ii) −*x*+1, *y*+1/2, −*z*+1/2; (iii) −*x*+3/2, −*y*, *z*+1/2.
